# Repeatability and reproducibility study of radiomic features on a phantom and human cohort

**DOI:** 10.1038/s41598-021-81526-8

**Published:** 2021-01-21

**Authors:** A. K. Jha, S. Mithun, V. Jaiswar, U. B. Sherkhane, N. C. Purandare, K. Prabhash, V. Rangarajan, A. Dekker, L. Wee, A. Traverso

**Affiliations:** 1 Department of Nuclear Medicine and Molecular Imaging, Tata Memoria Hospital, Mumbai, India; 2 Department of Medical Oncology, Tata Memoria Hospital, Mumbai, India; 3 Homi Bhabha National Institute (HBNI), Deemed University, Mumbai, India; 4 Department of Radiation Oncology (Maastro), GROW School for Oncology, Maastricht University Medical Centre+, Maastricht, The Netherlands

**Keywords:** Cancer, Oncology, Mathematics and computing, Image processing, Machine learning

## Abstract

The repeatability and reproducibility of radiomic features extracted from CT scans need to be investigated to evaluate the temporal stability of imaging features with respect to a controlled scenario (test–retest), as well as their dependence on acquisition parameters such as slice thickness, or tube current. Only robust and stable features should be used in prognostication/prediction models to improve generalizability across multiple institutions. In this study, we investigated the repeatability and reproducibility of radiomic features with respect to three different scanners, variable slice thickness, tube current, and use of intravenous (IV) contrast medium, combining phantom studies and human subjects with non-small cell lung cancer. In all, half of the radiomic features showed good repeatability (ICC > 0.9) independent of scanner model. Within acquisition protocols, changes in slice thickness was associated with poorer reproducibility compared to the use of IV contrast. Broad feature classes exhibit different behaviors, with only few features appearing to be the most stable. 108 features presented both good repeatability and reproducibility in all the experiments, most of them being wavelet and Laplacian of Gaussian features.

## Background

Medical images are routinely used for cancer staging, treatment planning and evaluation. Radiological findings are mainly evaluated in a qualitative or semi-qualitative fashion guided predominantly by visual inspection^1^. However, human interpretation of images is open to subjectivity and potentially misses some of the quantitative and objective information that could otherwise be retrieved from patients’ scans through computer-assisted methods^2^.

The field of “radiomics” aims to address the above-mentioned issues by objectively quantifying visual information in the images as a vast set of numerical metrics known as “features”. Radiomics hypothesizes that a certain subset of features, analyzed with the aid of machine learning algorithms due to high dimensionality, may have some predictive/prognostic value. Such subsets of features denote a “signature”, i.e. a digital image phenotype of the target disease, which opens the way towards personalized treatment in oncology^3^.

One of the most challenging problems for translating radiomic studies into clinical decision support systems is to evaluate the robustness of radiomic-based models and hence their potential generalizability across multiple datasets from different institutions^4^. Different institutions commonly acquire scans with different settings (e.g. scanner manufacturers, slice thickness, signal-to-noise ratio) according to largely self-defined imaging protocols, which add unwanted variation in the resulting radiomic features that are not related to the disease phenotype. A feature that is useful on one dataset may therefore lose its value on another dataset, since the feature may be sensitive to different methods of acquisition^5^.

When discussing robustness of radiomic studies two concepts need to be considered: “repeatability” and “reproducibility”. Repeatability refers to features that remain the same when imaged multiple times in the same subject, be that a human or a suitable phantom, using the same image acquisition methods. Reproducibility refers to features that remain the same when extracted using different equipment, different software, different image acquisition settings, or different operators (e.g. other clinics), be that in the same subject or in different subjects^6^. Repeatability and reproducibility concerns have been raised as major source of uncertainties in radiomic models^7^.

Most of the studies that investigated the reproducibility of radiomic features with respect to different image acquisition settings, demonstrate a strong dependence of radiomic features on such settings. Texture features appear to be more vulnerable to reproducibility/repeatability issues. There is a strong connection between reproducibility/repeatability and prognostic values^8^. In a study about time series classification, the investigators concluded that poorly reproducible/repeatable features were usually accompanied by poor discriminative performances^9^.

Recent publications have also investigated the presence of correlations between radiomic features and tumor volume^9,10^. The latter has been shown to be one of the most generalizable features. Therefore, there is the need to investigate if the most reproducible features were also strongly correlated with tumor volume.

Several studies have investigated the repeatability/ reproducibility of radiomic features on phantom as well as well as clinical cohort^6–9,10–17^. Few publications have also investigated the disease specific dependency of radiomic feature repeatability/ reproducibility and presented the results. These studies have either performed repeatability or reproducibility study alone; or performed repeatability and reproducibility study only on phantoms^13,14^ or clinical^15,16^ cohorts, which (1) limits the possibility to isolate a subset of features that are both repeatable and reproducible, and (2) does not allow comparing differences in the results because of using only phantom or human data. There remains a need to evaluate reproducibility and repeatability of radiomic features, not only on phantoms datasets, but also on human cohorts in the same study. The risk is that phantom studies do not have sufficiently high complexity and heterogeneity within the synthetic “tumors” to be a fair test of feature robustness. In our study, stable feature refers to both repeatable and reproducible features at the same time. With our study, we provide an extension to currently available literature by performing a comprehensive evaluation of the reproducibility and repeatability of 1080 radiomic features considering not only different groups of features, but also features extracted using digital filtering both with phantoms and human data. In this study, we also investigated how the correlations between radiomic features and tumor volume impact the reproducibility and repeatability results.

## Results

### Phantom—repeatability

**Figure 1:**
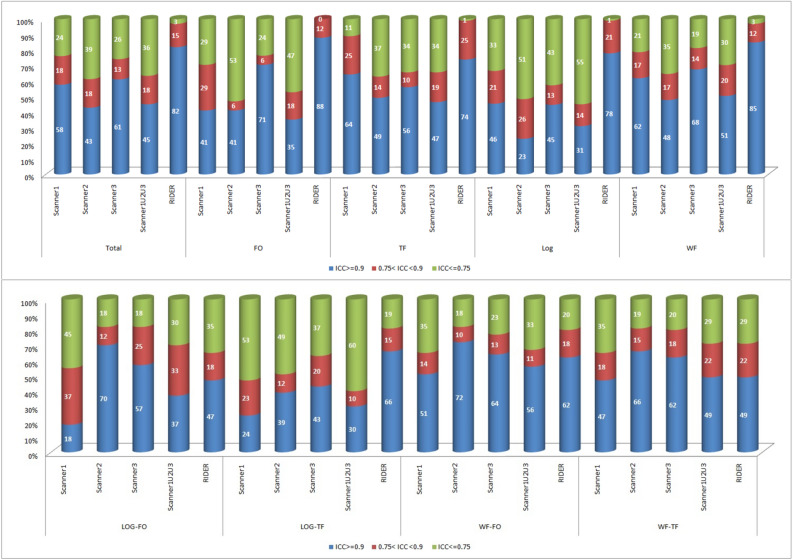
Repeatability analysis using repeated phantom scans for all the different radiomic feature classes. The median ICC values for all the 6 protocols is reported, separately for scanner1, scanner2, scanner3 and the union of the three. Repeatability analysis on RIDER (clinical cohort) was also performed. Three different levels of repeatability are defined: good (ICC ≥ 0.9), medium (0.75 < ICC < 0.9), and poor (ICC ≤ 0.75) (FO = First Order Feature; TF = Textural Feature; LOG = Total LoG Feature; WF = Total Wavelet Feature; LOG-FO = LoG First Order Feature; LOG-TF = LoG Textural Feature; WF-FO = Wavelet First Order Feature; WF-TF = Wavelet Textural Feature).

The percentage of radiomic features presenting good repeatability (ICC ≥ 0.9) were 58% (624/1080) for scanner1 (Philips Gemini TF16), 43% (464/1080) for scanner2 (Philips Gemini TF64), 61% (661/1080) for scanner3 (GE Discovery NM 570) and 45% (488/1080) for the three scanners overall. Results are shown in Fig. 1 for each feature category.

### RIDER (clinical cohort)—repeatability

The percentage of radiomic features presenting good, moderate, and poor repeatability were 82% (888/1080), 15% (164/1080), and 3% (28/1080) respectively for the RIDER clinical cohort. The results per feature categories are shown in Fig. 1.

### Phantom—reproducibility—intra and inter scanner variability

**Figure 2:**
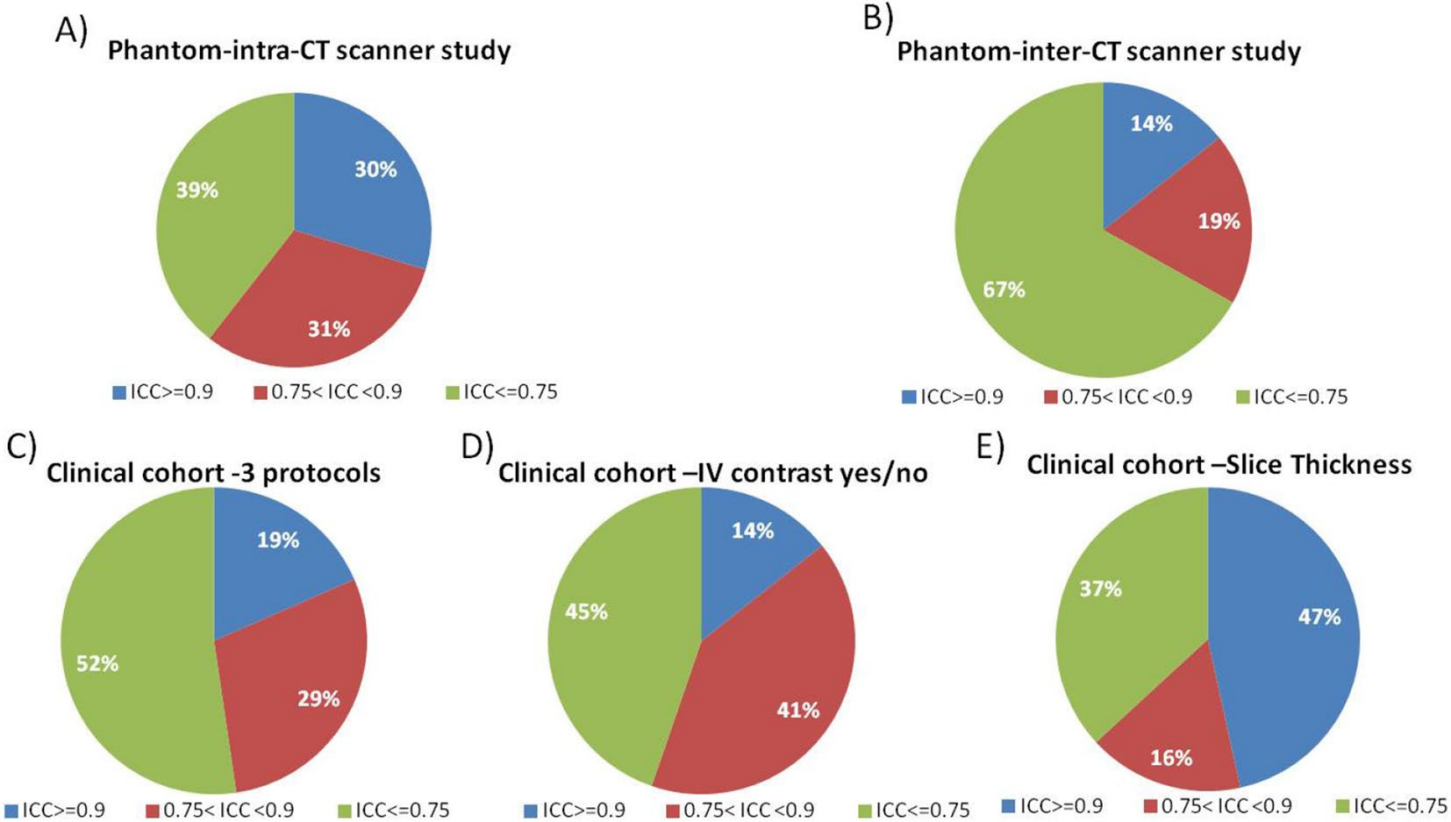
Results of the reproducibility experiments: (**A**) intra-scanner experiment using the phantom. By taking the median of all ICCs computed on the three scanners; (**B**) inter-scanner experiment using the phantom across all three scanners; (**C**) stability of radiomic features with respect to three different clinical protocols in the clinical study; (**D**) impact of IV (intravenous) contrast medium presence (WBCECT2)/ absence (NCCTT2) and difference in current (WBCECT2:Auto mA = 100–200; NCCTT2: fixed mA = 300 ) in the clinical study, and E) impact of slice thickness (2 vs 5 mm) in the human study. Three different levels of reproducibility are defined: good (ICC ≥ 0.9), medium (0.75 < ICC < 0.9), and poor (ICC ≤ 0.75).

For the intra-scanner study, 30% (322/1080), 31% (332/1080) and 39% (426/1080) features presented good, moderate, and poor reproducibility (Fig. 2A) for all the scanners. For the inter-CT scanner study, 14% (154/1080), 19% (204/1080) and 67% (722/1080) features presented good, moderate, and poor reproducibility respectively (Fig. 2B) for all the 6 protocols. Reproducibility of the features individually for the six protocols are shown in the Supplementary material S1.

### Clinical cohort—reproducibility

Among the features tested, 19% (199/1080) good, 29% (315/1080), moderate and 52% (556/1080) had poor, reproducibility when comparing the 3 different imaging protocols on the Gemini TF16 scanner (Fig. 2C).

When comparing IV contrast (WBCECT2) versus non-contrast (NCCTT2) protocols, 45% (483/1080) of the features had poor, 41% (442/1080) moderate, and 14% (155/1080) good reproducibility (Fig. 2D). When comparing slice thickness, using the BLDCT5 protocol (slice thickness = 5 mm) versus the WBCECT2 protocol (slice thickness = 2 mm), 37% (398/1080) of the features had poor, 17% (179/1080) moderate, and 47% (503/1080) good reproducibility (Fig. 2E).

### Volume correlations

In the clinical cohort, 7% (73/1080), 5% (57/1080) and 88% (950/1080) of the radiomic features had good (ρ ≥ 0.9), moderate (0.75 < ρ < 0.9) and poor (ρ ≤ 0.75) correlation with the GTV.

### Overall summary

**Figure 3:**
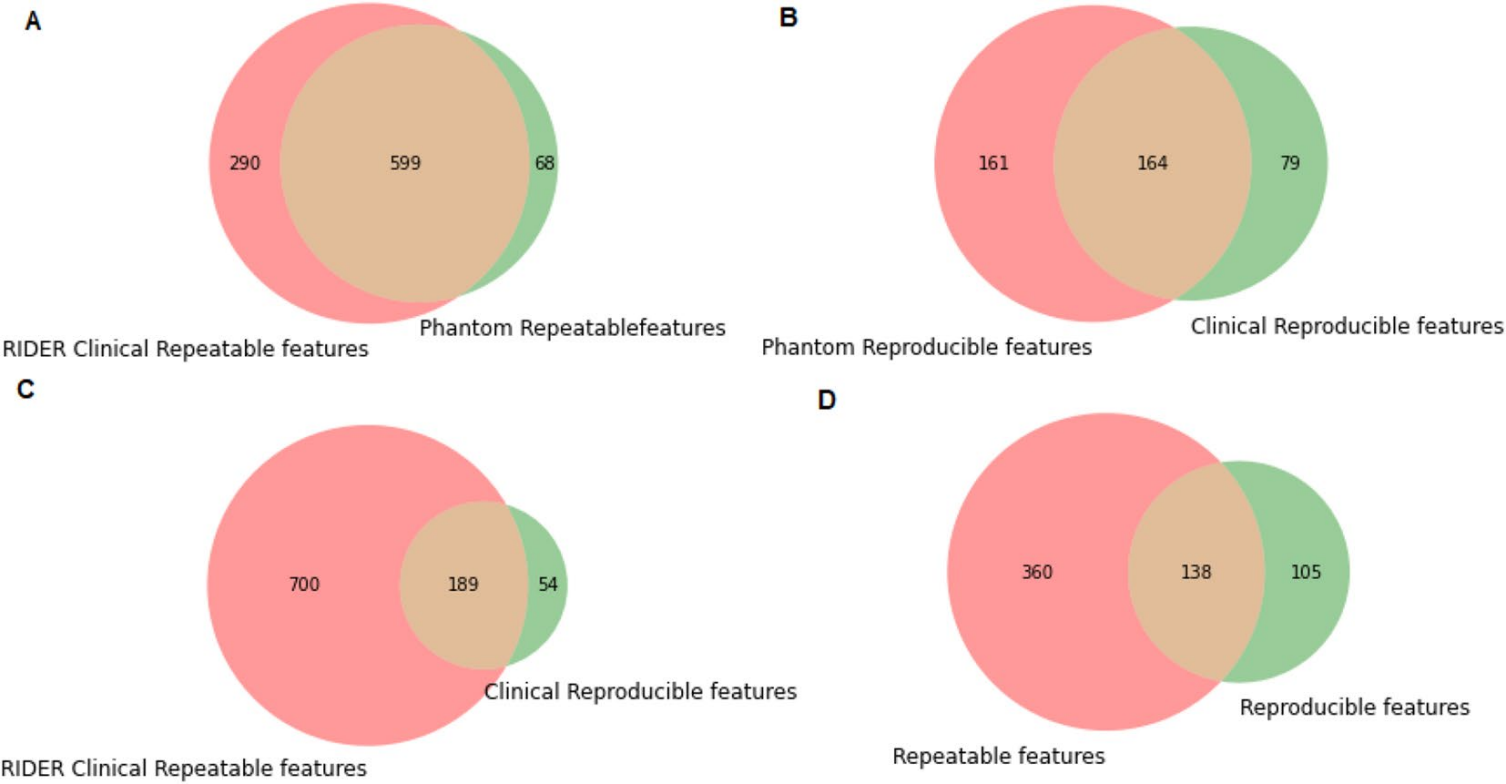
Common features in various studies showing good stability (ICC > 0.9): (**A**) Venn diagram shows the overlap of repeatability (RIDER) study and Phantom repeatability study. (**B**) Venn diagram shows the overlap of Phantom reproducibility study and reproducibility study in clinical cohort. (**C**) Venn diagram shows the overlap of repeatability (RIDER) study and reproducibility study in clinical cohort. (**D**) Overall summary of all the experiments. The Venn diagram shows the overlap of the repeatability experiment (phantom + clinical [RIDER] data) with the reproducibility experiments (phantom + clinical data).

Median ICC was calculated for all the reproducibility studies performed using the phantom and clinical cohorts. A total of 22.5% (243/1080) features had good reproducibility (ICC > 0.9) in clinical cohort. When the median of ICC was calculated for repeatability study performed with phantom and clinical cohorts (RIDER); 46.1% (498/1080) of features had good repeatability (ICC > 0.9). For repeatability study on phantom and clinical cohort together 55% (599/1080) features had good stability (ICC > 0.9) (Fig. 3A). For reproducibility study on phantom and clinical cohort together 15% (164/1080) features had good stability (ICC > 0.9) (Fig. 3B). For repeatability and reproducibility study together on clinical cohort 18% (189/1080) features had good stability (Fig. 3C). For all the experiments, 13% (138/1080) of the features presented both high (median ICC > 0.9) repeatability and high reproducibility (Fig. 3D). Tumor volume was again confirmed to be the most repeatable and reproducible feature with a median ICC of 0.99. When considering volume collinearity, 21% of these stable features presented strong Spearman correlations (ρ > 0.9). If we removed the features with strong correlations with GTV, then the final number of repeatable and reproducible features was 108: 59 WF (Wavelet) (8% of total WF), 46 LOG (Laplacian of Gaussian) (17% of total LOG), and 3 TA (Texture Analysis) (3% of total TA) features (Table 1). Overall, TA had the largest median ICC (0.933 ± 0.024) followed by LOG (0.923 ± 0.017) and WF (0.917 ± 0.014) features (*p* < 0.05). The topmost robust feature per feature types were: GLRLM-Non-Uniformity (LOG-2 mm kernel); LLH-GLCM-JointEnergy (WF) and Gray Level Dependence Matrix (GLDM) Non-Uniformity (TA).

**Table 1:** Overall summary of the 108 most repeatable and reproducible features for all the experiments and presenting correlations with tumour volume ρ < 0.9.

Overall summary TOP 108 features: 46 LOG, 59 WF, 3 TA					
Median ICC and STD per categories					
TA (0.933 ± 0.024)					
LOG (0.923 ± 0.017)					
WF (0.917 ± 0.014)					
Feature name	Category	Median ICC	Feature name	Category	Median ICC
**RANK 1–50**					
wavelet-LLH_glcm_JointEnergy	WF	0.9822	log-sigma-2**-**0-mm-3D_glrlm_ShortRunEmphasis	LOG	0.9295
log-sigma-2-0-mm-3D_glrlm_RunLengthNonUniformity	LOG	0.9766	wavelet-LLH_glrlm_ShortRunEmphasis	WF	0.9293
log-sigma-3-0-mm-3D_glrlm_RunLengthNonUniformity	LOG	0.9724	log-sigma-2**-**0-mm-3D_glrlm_RunLengthNonUniformityNormalized	LOG	0.9284
original_gldm_DependenceNonUniformity	TA	0.9571	log-sigma-2**-**0-mm-3D_firstorder_Entropy	LOG	0.9284
wavelet-LLH_glcm_Idm	WF	0.9542	wavelet-LHL_firstorder_10Percentile	WF	0.9280
log-sigma-3-0-mm-3D_firstorder_10Percentile	LOG	0.9484	log-sigma-1**-**0-mm-3D_gldm_LargeDependenceEmphasis	LOG	0.9274
log-sigma-3-0-mm-3D_glrlm_RunPercentage	LOG	0.9475	wavelet-LHL_firstorder_InterquartileRange	WF	0.9270
wavelet-LLH_glcm_Id	WF	0.9474	wavelet-LHL_firstorder_MeanAbsoluteDeviation	WF	0.9258
wavelet-LLH_glcm_SumEntropy	WF	0.9424	log-sigma-1**-**0-mm-3D_glrlm_ShortRunEmphasis	LOG	0.9257
log-sigma-3-0-mm-3D_firstorder_Mean	LOG	0.9409	wavelet-LHL_firstorder_RobustMeanAbsoluteDeviation	WF	0.9255
log-sigma-3-0-mm-3D_firstorder_Median	LOG	0.9386	wavelet-LHL_glcm_Imc2	WF	0.9252
log-sigma-2-0-mm-3D_glrlm_LongRunEmphasis	LOG	0.9379	wavelet-LHL_glcm_Idn	WF	0.9227
log-sigma-2-0-mm-3D_gldm_LargeDependenceEmphasis	LOG	0.9374	wavelet-LHL_glszm_SizeZoneNonUniformity	WF	0.9222
log-sigma-2-0-mm-3D_firstorder_10Percentile	LOG	0.9372	log-sigma-3**-**0-mm-3D_firstorder_MeanAbsoluteDeviation	LOG	0.9219
wavelet-LLH_glrlm_GrayLevelNonUniformityNormalized	WF	0.9367	wavelet-LHL_glszm_ZonePercentage	WF	0.9216
log-sigma-2-0-mm-3D_glrlm_RunVariance	LOG	0.9364	log-sigma-2**-**0-mm-3D_firstorder_Kurtosis	LOG	0.9213
log-sigma-2-0-mm-3D_glrlm_RunPercentage	LOG	0.9361	wavelet-LHL_gldm_SmallDependenceEmphasis	WF	0.9209
log-sigma-3-0-mm-3D_glrlm_RunLengthNonUniformityNormalized	LOG	0.9352	log-sigma-2**-**0-mm-3D_glcm_SumEntropy	LOG	0.9200
original_glrlm_RunLengthNonUniformity	TA	0.9333	log-sigma-2**-**0-mm-3D_firstorder_RobustMeanAbsoluteDeviation	LOG	0.9198
log-sigma-3-0-mm-3D_gldm_DependenceVariance	LOG	0.9326	log-sigma-3**-**0-mm-3D_firstorder_Kurtosis	LOG	0.9196
wavelet-LLH_glrlm_RunLengthNonUniformityNormalized	WF	0.9322	wavelet-LHL_ngtdm_Contrast	WF	0.9188
log-sigma-3-0-mm-3D_glrlm_ShortRunEmphasis	LOG	0.9317	wavelet-LHL_ngtdm_Strength	WF	0.9182
log-sigma-3-0-mm-3D_firstorder_RootMeanSquared	LOG	0.9314	wavelet-HLL_firstorder_10Percentile	WF	0.9174
wavelet-LLH_glrlm_RunPercentage	WF	0.9304	wavelet-HLL_firstorder_Entropy	WF	0.9169
log-sigma-2-0-mm-3D_firstorder_Mean	LOG	0.9297	wavelet-HLL_firstorder_InterquartileRange	WF	0.9169
**RANK 51–100**					
wavelet-HLL_firstorder_MeanAbsoluteDeviation	WF	0.9168	wavelet-HLL_gldm_LargeDependenceEmphasis	WF	0.9089
wavelet-HLL_firstorder_RobustMeanAbsoluteDeviation	WF	0.9167	wavelet-HLL_gldm_SmallDependenceEmphasis	WF	0.9089
log-sigma-2-0-mm-3D_glcm_JointEntropy	LOG	0.9163	original_gldm_SmallDependenceEmphasis	TA	0.9084
wavelet-HLL_firstorder_Uniformity	WF	0.9162	log-sigma-2**-**0-mm-3D_glcm_JointEnergy	LOG	0.9082
wavelet-HLL_glcm_DifferenceAverage	WF	0.9157	wavelet-HLL_ngtdm_Contrast	WF	0.9074
wavelet-HLL_glcm_DifferenceEntropy	WF	0.9154	wavelet-HHL_glrlm_RunPercentage	WF	0.9073
log-sigma-3-0-mm-3D_firstorder_Variance	LOG	0.9153	wavelet-HHL_gldm_LargeDependenceEmphasis	WF	0.9069
log-sigma-2-0-mm-3D_firstorder_RootMeanSquared	LOG	0.9142	log-sigma-3**-**0-mm-3D_firstorder_RobustMeanAbsoluteDeviation	LOG	0.9059
log-sigma-2-0-mm-3D_firstorder_Uniformity	LOG	0.9139	wavelet-LLL_firstorder_Entropy	WF	0.9056
log-sigma-2-0-mm-3D_glcm_Id	LOG	0.9138	log-sigma-1**-**0-mm-3D_firstorder_RobustMeanAbsoluteDeviation	LOG	0.9055
log-sigma-2-0-mm-3D_glcm_Idm	LOG	0.9137	wavelet-LLL_firstorder_RootMeanSquared	WF	0.9051
wavelet-HLL_glcm_JointEntropy	WF	0.9134	wavelet-LLL_glcm_Contrast	WF	0.9051
wavelet-HLL_glcm_Idm	WF	0.9132	wavelet-LLL_glcm_DifferenceAverage	WF	0.9044
wavelet-HLL_glcm_Idmn	WF	0.9131	log-sigma-3**-**0-mm-3D_firstorder_InterquartileRange	LOG	0.9042
wavelet-HLL_glcm_Id	WF	0.9131	log-sigma-2**-**0-mm-3D_glrlm_GrayLevelNonUniformityNormalized	LOG	0.9039
wavelet-HLL_glcm_Idn	WF	0.9130	log-sigma-2**-**0-mm-3D_glcm_DifferenceAverage	LOG	0.9039
wavelet-HLL_glcm_MaximumProbability	WF	0.9123	wavelet-LLL_glcm_DifferenceEntropy	WF	0.9037
log-sigma-2-0-mm-3D_firstorder_MeanAbsoluteDeviation	LOG	0.9120	wavelet-LLL_glcm_JointEntropy	WF	0.9036
wavelet-HLL_glcm_SumEntropy	WF	0.9119	log-sigma-2**-**0-mm-3D_glcm_DifferenceEntropy	LOG	0.9036
log-sigma-1-0-mm-3D_glrlm_RunPercentage	LOG	0.9115	log-sigma-1**-**0-mm-3D_glcm_Id	LOG	0.9035
wavelet-HLL_glrlm_GrayLevelNonUniformityNormalized	WF	0.9114	wavelet-LLL_glcm_Idm	WF	0.9032
wavelet-HLL_glrlm_RunLengthNonUniformityNormalized	WF	0.9112	log-sigma-1**-**0-mm-3D_glrlm_RunLengthNonUniformityNormalized	LOG	0.9031
log-sigma-2-0-mm-3D_firstorder_InterquartileRange	LOG	0.9110	wavelet-LLL_glcm_Id	WF	0.9031
wavelet-HLL_glrlm_RunPercentage	WF	0.9109	log-sigma-1**-**0-mm-3D_firstorder_InterquartileRange	LOG	0.9026
wavelet-HLL_glrlm_RunVariance	WF	0.9107	wavelet-LLL_glcm_Idn	WF	0.9025
wavelet-HLL_glrlm_ShortRunEmphasis	WF	0.9105	log-sigma-1**-**0-mm-3D_firstorder_10Percentile	LOG	0.9015
wavelet-HLL_glszm_LargeAreaEmphasis	WF	0.9098	wavelet-LLL_gldm_DependenceNonUniformity	WF	0.9009
wavelet-HLL_glszm_ZonePercentage	WF	0.9098	wavelet-LLL_gldm_DependenceNonUniformityNormalized	WF	0.9005
wavelet-HLL_glszm_ZoneVariance	WF	0.9092	wavelet-LLL_gldm_SmallDependenceEmphasis	WF	0.9001

The features are ordered by decreasing median ICC values (computed on all the experiments). Most reproducible and repeatable features per categories were: GLRLM-Non-Uniformity (LOG-2 mm kernel); LLH-GLCM-JointEnergy (WF) and Gray Level Dependence Matrix (GLDM) Non-Uniformity (TA).

**Figure 4:**
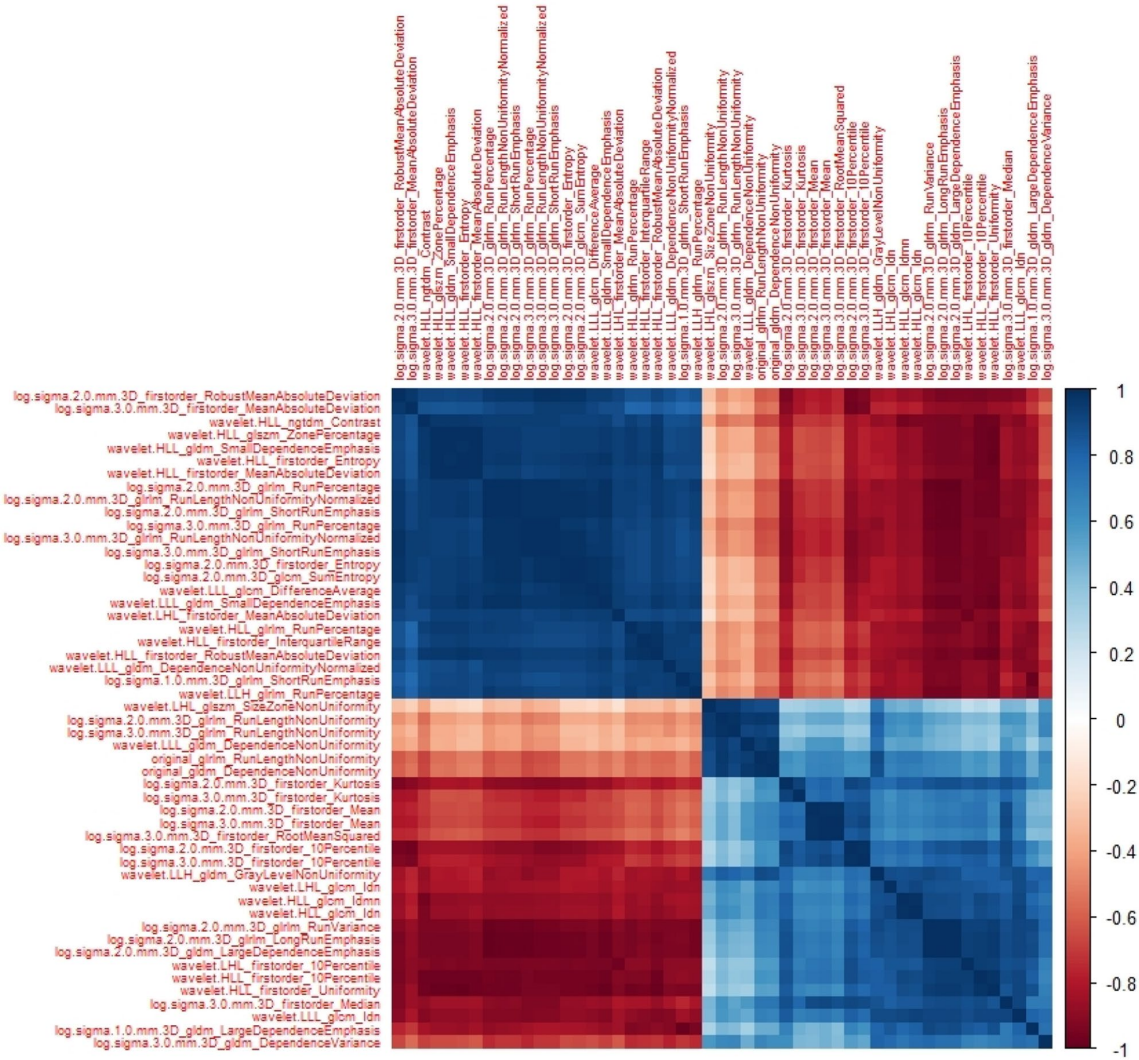
Heatmap showing Spearman correlations between the top 50 repeatable features.

It is interesting to notice how the top 50 repeatable features presented strong inter Spearman correlations, with Wavelet and Laplacian of Gaussian features being strongly clustered together (heatmap on Fig. 4). Overall, the number of features with good repeatability was found to be significantly larger than the number of reproducible features. Reproducibility experiments using phantom data (IntraCT experiment) led to more features being found reproducible compared to experiments performed using the clinical cohort (30% vs 19% of features with ICC ≥ 0.9, *p* < 0.05). Around 57% (138/243) of the robust features overlapped with features from repeatability and reproducibility study. The remaining 67 features being 36% Wavelet and 74% Laplacian of Gaussian were reproducible, but not repeatable.

## Discussion

In this study, we investigated: (A) radiomic feature repeatability in a test–retest scenario using a NEMA IQ phantom; (B) radiomic feature reproducibility with respect to different tube currents, slice thickness as well as dependencies to different scanner models using an image quality phantom, and (C) radiomic feature reproducibility in a clinical cohort comparing three different acquisition protocols as well as the impact of slice thickness and the presence of IV contrast medium. We isolated a list of repeatable and reproducible features for all the experiments. Furthermore, we computed the correlations between radiomic features and tumor volume with the aim of investigating if the most repeatable and reproducible features also presented strong correlations. In fact, tumor volume was found to be the most robust feature and we wanted to assess if this could be a reason for a feature to present high reproducibility and repeatability. As shown in the results, only a relatively small percentage of radiomic features (around 13% of the total) presented both good repeatability and reproducibility across all the experiments. However, differences were found between repeatability and reproducibility. The number of features with good repeatability was larger than the number of reproducible features in the phantom experiment. Unfortunately, because we did not have any test–retest clinical data it was not possible to draw the same conclusion. Nevertheless, to obtain a fair comparison, we used the publicly available dataset RIDER to investigate the repeatability of radiomic features in NSCLC patients. The Venn Diagram in Fig. 3D shows that most of the repeatable and reproducible features in human data overlap with features from the phantom studies. This clearly shows that features computed on phantom are a superset of features computed on real human data. Our experiments also showed that there are some features extracted from human data that are robust but do not overlap with phantom results. Two main reasons could be associated with this: (A) statistical fluctuations because of the large number of computed features; (B) differences in the dynamic range of the features between phantom and human data. Point (B) is strictly related to the fact that the image quality phantom with spherical homogenous inserts are still not advanced enough to replicate tumor complexity seen in patients’ data. Therefore, our study should be improved by including several types of imaging phantoms or considering new types of plugs that can better mimic tumor heterogeneity. In the last years, attention has been devoted to produce more realistic inserts by using 3D printing techniques^18,19^. The above-mentioned hypothesis seem to be confirmed by the fact that the features that did not overlap were only wavelet and Laplacian of Gaussian features, which might indicate that some real tumors’ texture patterns are still difficult to be reproduced with imaging phantoms.

We found large variation of radiomic feature in repeatability study even within a short time gap of 30 min “coffee-break”. Overall, less than 50% of features had a good repeatability (ICC > 0.9) using phantom scans, in agreement with previously published literature^19–21^. When considering time-series analysis of radiomic features (e.g. for monitoring treatment response), temporal stability of radiomic features becomes imperative to be investigated. As mentioned in the introduction section, poor repeatability seems to be associated with poor prognostic/predictive power, while the reverse might not be equally true^9^. Therefore, our results can be taken by other radiomic studies to reduce the dimensionality of computed features by excluding poorly repeatable features.

When considering radiomic reproducibility, the presence or absence of IV contrast medium had a stronger impact than differences in slice thickness in the human study: 14% (155/1080) versus 47% (503/1080) (*p* < 0.05) of features with good reproducibility.

From the overall summary section in the results, it emerges that the different feature categories are sensitive with different degrees to reproducibility and repeatability. Our results are in line with the previous literature. The usage of image filtering could enhance the quality of the images even when acquired with different protocols and thus improve reproducibility. It is important to point out that this study did not investigate the robustness of shape metrics, since the contours were co-registered from PET to CT images and the same contour was used for all sets of CT series. However, shape metrics have been shown to be strongly affected by inter-observer variability in tumor delineations and this aspect was not investigated in this study.

We investigated how correlations between tumor volume and radiomic features could impact the repeatability and reproducibility. In line with other studies, not only tumor volume was the most repeatable and reproducible feature (median ICC = 0.99), but most of the top reproducible features showed strong Spearman correlations (ρ > 0.9) with tumor volume. This opens the debate whether their robustness could be an effect of an underlying “volume effect”. However, more investigation is needed to isolate and further explain this effect. Therefore, in Table 1 we proposed the final list of most repeatable and reproducible features with lower correlations with tumor volume.

Finally, the list provided in Table 1 represents a starting point to isolate repeatable and robust features, but this is not enough to conclude about their prognostic predictive performance. Furthermore, as shown in Fig. 4, most of these features present strong intercorrelations and might produce redundant information if all are injected into a classifier for radiomic-based models. The results presented in this study needs to be validated in additional multi-institutional studies and considering additional parameters that can affect features’ reproducibility and repeatability. First, in our analysis we only considered two different scanner manufacturers. We did not investigate the role of other acquisition parameters such as reconstruction kernels or tube voltage. These results are intended to be shared within the radiomic community for confirmation.

## Methods

This study was approved by the hospital Institutional Ethics Committee (Institutional Ethics Committee-I, Tata Memorial Centre [IEC, TMC], Mumbai, India) as a retrospective study, with waivers of informed consent from involved patients as per IEC policy of our hospital by the same Ethics Committee. All methods were carried out in accordance with relevant guidelines and regulations. This study comprises PET/CT images from a polymer phantom as well as from a clinical cohort. Our study has focused only on CT radiomic features stability. PET images were used to delineate the tumor (using SUV threshold of 40%) and this delineation was transferred to the corresponding CT images included in this study.

### Phantom

The National Electrical Manufacturers Association (NEMA) Image Quality (IQ)PET/CT phantom (Data Spectrum Inc., NJ, USA) was used for this study^22^. The external dimensions of the phantom are 241 mm × 305 mm × 241 mm with interior length of 180 mm and volume of 9.7L. It has six fillable spheres and one central cylinder. The largest insert with a diameter of 37 mm was used for radiomic feature analysis study. The phantom was filled with distilled water containing 18F-FDG. The concentration of 18F-FDG was adjusted until a target to background signal ratio of 4:1 was created between the active sphere and water background.

### Clinical cohort

Patients with non-small cell lung cancer (NSCLC) (n = 104) who underwent pre-treatment PET/CT scans in our department were included in this study. There were 85 males and 19 females. The median age was 66 (36–90) and 53 (35–72) years respectively for males and females. The median tumor volume was 92 (14–486) cm^3^ for men and 86 (22–432) cm^3^ for women. Population demographics and clinical information are provided in Supplementary table S2.

RIDER: The Reference Image Database to Evaluate Therapy Response (RIDER) data base was used in this study to perform repeatability study. All the 32 patients DICOM data (i.e. Images and RTSTRUCTs) of the RIDER data set were included in this study^23^.

### Scanners

Three different scanners were used in the study. Two scanners were from the same manufacturer (Philips Medical, Eindhoven, The Netherlands) but different models, and the last scanner was from another manufacturer (General Electric Medical System, Milwaukee, USA). For simplicity of reading we will refer to the scanners as follows: scanner 1 is the Philips Gemini TF16 PET/CT, scanner 2 is the Gemini TF64 PET/CT, and scanner 3 is the General Electric Discovery NM 670 pro SPECT/CT.

#### Scanning protocols

##### NEMA IQ phantom

The NEMA IQ phantom was scanned twice, 30 min apart (‘coffee break’) without repositioning, one the same scanner and within the same conditions. This procedure was performed for all the three scanners and considering six different acquisition protocols. They had the same tube voltage (120 kV for all three scanners), pitch (0.46 for scanner 1 and 2 and 2.5 for Scanner 3) and reconstruction kernel based on filtered back projection for scanner 1, 2 and adaptive statistical iterative reconstruction (ASiR) (40% ASiR setting and a noise index of 13.75) for scanner 3, but different tube currents (ranging from 100 to 300 mA) slice thicknesses (ranging from 2 to 5 mm for scanner 1& 2 and 2.5 to 5 for scanner 3). These protocols are listed in Table 2.

**Table 2:** Overview of the scanning protocols used to acquire images with the IQ phantom.

Protocol name	Tube current (mA)	Reconstruction slice thickness (mm) [voxel size (cubic millimeter)] (scanner 1&2)	Reconstruction slice thickness (mm) [voxel size (cubic millimeter)] (scanner 3)
Protocol 1	100	2 [0.86 × 0.86 × 2]	2.5 [0.9653 × 0.9653 × 2.5]
Protocol 2	100	5 [0.86 × 0.86 × 5]	5 [0.9653 × 0.9653 × 5]
Protocol 3	200	2 [0.86 × 0.86 × 2]	2.5 [0.9653 × 0.9653 × 2.5]
Protocol 4	200	5 [0.86 × 0.86 × 5]	5 [0.9653 × 0.9653 × 5]
Protocol 5	300	2 [0.86 × 0.86 × 2]	2.5 [0.9653 × 0.9653 × 2.5]
Protocol 6	300	5 [0.86 × 0.86 × 5]	5 [0.9653 × 0.9653 × 5]

Six scanning protocols, with same tube voltage (120 kV), pitch (Scanner 1&2: 0.46; Scanner 3: 2.5), and reconstruction kernel, but different tube currents and slice thicknesses were investigated. The phantom was scanned twice on scanners 1–2–3 without repositioning in a 30-min test–retest scenario. The total number of scans acquired with the IQ phantom is 6 protocols × 3 scanners × 2 (test–retest) = 36 scans.

##### Clinical cohort

Patients were scanned using three different clinical protocols on the Philips Gemini TF64 PET/CT (previously referred to as scanner 2). The three protocols had the same tube voltage (120 kV), pitch (0.46) and reconstruction kernel, but different slice thicknesses, tube current and presence or absence of an intravenous contrast medium, namely, one whole body contrast CT with 2 mm slice thickness (referred as WBCECT2), one whole body contrast CT with 5 mm slice thickness (referred as BLDCT5), and one non contrast thoracic CT with 2 mm slice thickness (referred as NCCTT2). Modulated tube current (between 100 and 200 mA) as per dose care automated system was used for BLDCT5 and WBCECT2. The protocols are listed in Table 3.

**Table 3:** Overview of the clinical protocols. Images were acquired on the Philips Gemini TF64 PET/CT (previously referred to as scanner 2) with three different protocols.

Clinical protocol name	Slice thickness (mm)	Intravenous contrast medium	Tube current (mA)	Voxel size (cubic millimeters)
BLDCT5	5	Yes—nonionic contrast	Modulated auto-mA (100–200)	1.17 × 1.17 × 5
WBCECT2	2	Yes—nonionic contrast	Modulated auto-mA (100–200)	1.17 × 1.17 × 2
NCCTT2	2	NO	Fixed mA 300	0.87 × 0.87 × 2

#### RIDER

The RIDER data set comprises of 32 NSCLC patient’s test–retest CT imaging performed with a time lag of 15 min and two sets of delineations (RTSTRUCT) (i.e. tumor delineated by manual and automatic methods). Imaging parameters of RIDER database is summarized in Table 4. Radiomic extraction and statistical analysis was performed as per the study protocol.

**Table 4:** The imaging protocol of the RIDER data set.

Parameters	Rider data set
Manufacturer	GE healthcare
Acquisition type	Helical
Tube voltage	120 kVp
Tube current	Range 165–549 mAs
Slice thickness	1.25 mm
Pixels	512 × 512
Voxel size (cubic millimeter)	0.66 × 0.66 × 1.25

#### Study design

**Figure 5:**
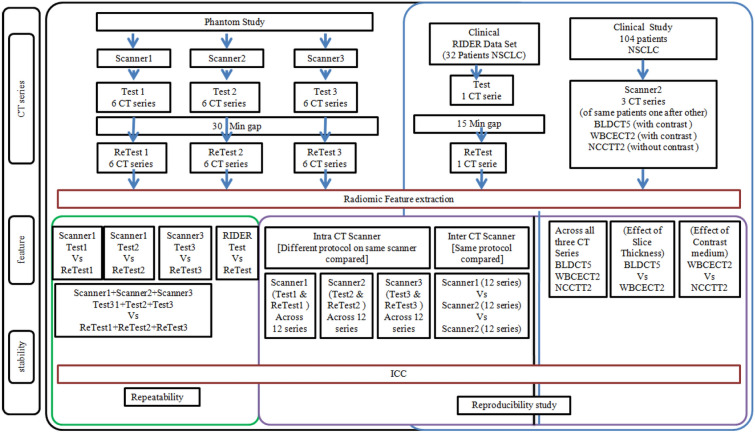
In this study we investigated both reproducibility and repeatability of radiomic features. The repeatability of radiomic features was evaluated using the test retest scans acquired with the IQ phantom on three different scanners and with 6 protocols and online available RIDER data set. The reproducibility of radiomic features with respect to different acquisition protocols but within the same scanner (intra-scanner variability) was evaluated comparing radiomic feature values using the test–retest scans acquired with the IQ phantom across the 6 different protocols. A clinical cohort of NSCLC patients was used to investigate the reproducibility of radiomic features with respect to 3 different clinical acquisition protocols, with a focus on the impact of slice thickness and IV contrast medium.

In this study we investigated both reproducibility and repeatability of radiomic features. The repeatability of radiomic features was evaluated using the test retest scans acquired with the IQ phantom on three different scanners and for all the 6 protocols listed in Table 2 and on the publicly available clinical cohort RIDER data set. The reproducibility of radiomic features with respect to different acquisition protocols but within the same scanner (intra-scanner variability) was evaluated comparing radiomic feature values using the test scans acquired with the IQ phantom across the 6 different protocols. This analysis was repeated for all the three scanners. The reproducibility of radiomic features with respect to different scanner models was evaluated comparing radiomic feature values extracted from the test scans acquired with the IQ phantom for each protocols on the three different scanners (inter-scanner variability). The reproducibility of radiomic features with respect to presence/absence of intravenous contrast medium and slice thickness in clinical data was investigated comparing radiomic features using the images acquired with the NSCLC patients (clinical study). Figure 5 summarizes the overall study design.

##### ROIs (Region of Interest) definition

PET and CT series of all the studies were loaded on a GE Advantage image processing workstation (GE Healthcare, Waukesha, WI, USA) from our hospital PACS. Standardized Uptake Value (SUV)-based auto-segmentation using a threshold of 40% from the maximum value was used to delineate the primary lung tumor and active phantom insert on PET images for scanners 1 and 2. Manual delineation of the phantom insert was performed by an experienced physicist for phantom images acquired with scanner 3, since PET series were not available for this scanner. These delineations were performed using the AdvantageSimMD software installed on the Advantage image processing workstation and stored as RTSTRUCT. This RTSTRUCT creates a ROI instance corresponding to each PET and CT series in the study^24^. As all the PET and CT series belongs to same study it automatically accounts for differences in resolution between PET and CT images when the RTSTRUCT is saved. The stored RTSTRUCT has the location of the ROI instance for corresponding image sets (series) about matrix size and slice thickness of that series. Images and ROIs, in form of DICOM and RTSTRUCT files, respectively, were transferred to a research workstation where radiomic features were extracted.

##### Image pre-processing

Images and ROIs are saved in Digital Imaging and Communications in Medicine (DICOM) format. However, the Pyradiomics software uses images and ROIs in Nearly Raw Raster Data (NRRD) format for radiomic feature extraction. We used an in-house developed python Script to perform batch processing to convert images and ROIs from a DICOM CT and RTSTRUCT into an NRRD format using 3DSlicer v4.10.2^25^. An in-house python script based on the image processing toolkit simpleITK v1.2.0 was used to convert contours to binary masks^26^.

All images were re-sampled to isotropic voxel of 2 × 2 × 2 cubic millimeters prior to 3D radiomic feature extraction using the default b-spline interpolation function in simpleITK. A fixed-bin width of 25 was used for grey level binning of the images. Radiomic features were extracted from the original CT images as well as from images with the following filters: (A) wavelet transformed images using the standard wavelets transforms implemented in Pyradiomics v2.2.0; (B) Laplacian of Gaussian with sigma values 1, 2 and 3 mm. All the features were extracted in 3D, with texture features were aggregated with the method 3D Average.

##### Radiomic feature definitions

The following radiomic features were extracted as per the definition provided in Pyradiomics documentation (https://pyradiomics.readthedocs.io/en/latest/): First Order Statistics (FO-17 features); Gray Level Co-Occurrence Matrix (GLCM-22 features); Gray Level Run Length Matrix (GLRLM-16 features); Gray Level Size Zone Matrix (GLSZM-16 features); Neighboring Gray Tone Difference Matrix (NGTDM-5 features); Gray Level Dependence Matrix (GLDM-14 features); plus corresponding features with Laplacian of Gaussian filters (LOG-270 features) and with wavelet (WF-720 features). A total of 1080 radiomic features were extracted.

##### Statistical analysis

The ICC (Intraclass Correlation Coefficient) based on a two-way mixed effect, consistency, single rater/measurement was used to measure the repeatability/reproducibility of features for our experiments, as per Eq. (1)^27^. Three different level of repeatability/reproducibility were defined: good (ICC ≥ 0.90); moderate (0.75 < ICC < 0.90); poor (ICC <  = 0.75)^28^.

1 $$ICC3 = \frac{{MS_{R} - MS_{E} }}{{MS_{R} + \left( {k - 1} \right)MS_{E} }}$$

Definition of the ICC used as reproducibility metric. Where, MS_E_ = mean square for error, MS_R_ = mean square for rows, k = number of raters/measurements.

For the repeatability experiment, the ICC was computed between test and re-test scans for all the 6 protocols, separately and together for all the scanners. For the repeatability study with the RIDER dataset, the ICC was computed between test and re-test scans. For the intra-scanner reproducibility experiment, ICC values were computed separately for the three scanners and the median ICC value is reported in the results. For the inter-scanner reproducibility experiment, the ICC values were computed comparing radiomic features separately for the six protocols between the three scanners. The median ICC values for the 6 protocols is reported. For the clinical study, the ICC values comparing radiomic features between the three protocols are reported, as well as only comparing protocols BLDCT5 versus NCCTT2 (same slice thickness but with and without intravenous contrast medium) and NCCTT2 versus WBCECT2 (both with intravenous contrast medium, but different slice thicknesses).

##### Commonality study

In the clinical cohort common good stable features were found between repeatability study of RIDER data set and reproducibility study of our clinical cohort. Median ICC of repeatability study (Phantom and clinical cohort [RIDER]) was as well as reproducibility study (Phantom and clinical cohort) was calculated. Median ICC of repeatability and Reproducibility study was compared to find common good (ICC > 0.9) stable features.

##### Volume collinearity analysis

Using the clinical cohort, we assessed the correlation between the GTV and radiomic features using the Spearman correlation coefficient (ρ) to account for possible nonlinear dependencies. The median Spearman correlation coefficient between the 3 different protocols is used in the analysis.

Statistical analysis was performed using R (version 3.2.3) using the package *psych.* *p* values were corrected for multiple comparisons using the false-discovery rate corrections method and statistical significance after correction was set at *p* < 0.05.

## Supplementary information

Supplementary Material S1

Supplementary Table S2

## References

[1] Beaton L, Bandula S, Gaze MN, Sharma RA (2019). How rapid advances in imaging are defining the future of precision radiation oncology. Br. J. Cancer.

[2] Gillies RJ, Kinahan PE, Hricak H (2016). Radiomics: images are more than pictures, they are data. Radiology.

[3] Gardin I (2019). Radiomics: principles and radiotherapy applications. Crit. Rev. Oncol. Hematol..

[4] Kumar V (2012). Radiomics: the process and the challenges. Magn. Reson. Imaging.

[5] Yip SSF, Aerts HJWL (2016). Applications and limitations of radiomics. Phys. Med. Biol..

[6] O’Connor JPB (2017). Imaging biomarker roadmap for cancer studies. Nat. Rev. Clin. Oncol..

[7] Traverso A, Wee L, Dekker A, Gillies R (2018). Repeatability and reproducibility of radiomic features: a systematic review. Int. J. Radiat. Oncol. Biol. Phys..

[8] Gudmundsson S, Runarsson TP, Sigurdsson S (2012). Test–retest reliability and feature selection in physiological time series classification. Comput. Methods Programs Biomed..

[9] Zhovannik I, Bussink J, Dekker A (2019). Volume bias in textural radiomics. Int. J. Radiat..

[10] van Griethuysen JJM (2017). Computational radiomics system to decode the radiographic phenotype. Can. Res..

[11] Larue RTHM, Defraene G, De Ruysscher D (2017). Quantitative radiomics studies for tissue characterization: a review of technology and methodological procedures. Br. J. Radiol..

[12] Kim HG, Chung YE, Lee YH (2015). Quantitative analysis of the effect of iterative reconstruction using a phantom: determining the appropriate blending percentage. Yonsei Med. J..

[13] Shafiq-Ul-Hassan M, Zhang GG, Latifi K (2017). Intrinsic dependencies of CT radiomic features on voxel size and number of gray levels. Med. Phys..

[14] van Timmeren JE, Leijenaar RTH, van Elmpt W, Wang J, Zhang Z, Dekker A, Lambin P (2016). Test-retest data for radiomics feature stability analysis: generalizable or study-specific?. Tomography.

[15] Balagurunathan Y, Gu Y, Wang H (2014). Reproducibility and prognosis of quantitative features extracted from CT images. Transl. Oncol..

[16] Parmar C, Rios Velazquez E, Leijenaar R (2014). Robust radiomics feature quantification using semiautomatic volumetric segmentation. PLoS ONE.

[17] Welcha ML, McIntoshe C, Haibe-Kainsa B (2019). Vulnerabilities of radiomic signature development: the need forsafeguards. Radiother. Oncol..

[18] Mitsouras D, Liacouras P, Imanzadeh A (2015). Medical 3D printing for the radiologist. Radiographics.

[19] Samei E, Hoye J, Zheng Y, Solomon JB, Marin D (2019). Design and fabrication of heterogeneous lung nodule phantoms for assessing the accuracy and variability of measured texture radiomics features in CT. J. Med. Imaging (Bellingham).

[20] Traverso A (2019). Stability of radiomic features of apparent diffusion coefficient (ADC) maps for locally advanced rectal cancer in response to image pre-processing. Phys. Med..

[21] Sanduleanu S (2018). Tracking tumor biology with radiomics: a systematic review utilizing a radiomics quality score. Radiother. Oncol..

[22] Jha AK, Mithun S, Puranik AD (2019). Performance characteristic evaluation of a bismuth germanate-based high-sensitivity 5-ring discovery image quality positron emission tomography/computed tomography system as per National Electrical Manufacturers Association NU 2–2012. World J. Nucl. Med..

[23] Armato SG, Meyer CR, Mcnitt-Gray MF, McLennan G, Reeves AP, Croft BY, Clarke LP, RIDER Research Group (2008). The Reference Image Database to evaluate response to therapy in lung cancer (RIDER) project: a resource for the development of change-analysis software. Clin. Pharmacol. Ther..

[24] CT/PET FUSION DICOM CONFORMANCE STATEMENT for DICOM V3.0, Technical Publications Direction 2290660-100 Revision A, GE Medical Systems. https://www.gehealthcare.com/-/jssmedia/5337d686cfe442b2a75083038a877029.pdf?la=en-us. Accessed 17 July 2020.

[25] Fedorov A (2012). 3D slicer as an image computing platform for the quantitative imaging network. Magn. Reson. Imaging.

[26] Lowekamp BC, Chen DT, Ibáñez L, Blezek D (2013). The design of simple ITK. Front. Neuroinform..

[27] Bartko JJ (1966). The intraclass correlation coefficient as a measure of reliability. Psychol. Rep..

[28] Koo TK, Li MY (2016). A guideline of selecting and reporting intraclass correlation coefficients for reliability research. J. Chiropract. Med..

