# Supplementary material for: Repeatability and reproducibility study of radiomic features on a phantom and human cohort
Source: Sci Rep. 2021 Jan 21;11:2055. doi: 10.1038/s41598-021-81526-8 (PMC7820018; doi:10.1038/s41598-021-81526-8)
Supplement: Supplementary file 1 — Supplementary Material S1 [file 41598_2021_81526_MOESM1_ESM.docx]

**Repeatability and reproducibility study of radiomic features on a phantom and human cohort**

A.K. Jha^1,3,4+^, S. Mithun^1,3,4^, V. Jaiswar^1^, U.B. Sherkhane^1^, N.C. Purandare^1,3^, K. Prabhash^2,3^, V. Rangarajan^1,2^, A. Dekker^4^, L. Wee^4*^ and A. Traverso^4*^

^1^ Department of Nuclear Medicine and Molecular Imaging, Tata Memoria Hospital, Mumbai, India

^2^ Department of Medical Oncology, Tata Memoria Hospital, Mumbai, India

^3^ Homi Bhabha National Institute (HBNI), Deemed University, Mumbai, India

^4^ Department of Radiation Oncology (Maastro), GROW School for Oncology, Maastricht University Medical Centre+, Maastricht, The Netherlands

^+^Corresponding author: A.K. Jha, ashish.kumar.jha.77@gmail.com

^*^These authors share senior authorship

Supplementary material S1:

Stability of radiomic features for all the six protocols of phantom study (Series description is given in table 2)
